# Decoding the cGAS–STING–eosinophils predictive and natural therapeutic molecular signature in burn injury progression and keloid formation: insights from artificial intelligence-driven multiomics

**DOI:** 10.3389/fsurg.2026.1846856

**Published:** 2026-05-29

**Authors:** Jingjing Li, Qinghua Yang

**Affiliations:** Department of Comprehensive Plastic Surgery, Plastic Surgery Hospital, Chinese Academy of Medical Sciences and Peking Union Medical College, Beijing, China

**Keywords:** artificial intelligence, burn injury, keloid formation, multiomics, predictive model

## Abstract

**Background:**

Inflammation-related pathway activation contributes to the progression of burn injury and keloid formation.

**Objective:**

Our study aimed to investigate the role of the cGAS–STING pathway in regulating eosinophils during the progression of burn injury and keloid formation.

**Methods:**

Key cGAS–STING pathway and eosinophils (CE)-related differentially expressed genes (DEGs) were identified in burn injury patient bulk profiles (GSE19743) using Limma, WGCNA, CIBERSORT, and STRING frameworks. Next, a systemic and explainable machine learning model was utilized to identify predictive model hub genes involved in keloid formation of burn injury patients (GSE37069) and burn-injury-associated keloid patient (GSE42270), based on CE-associated DEGs. The pathogenic role of hub genes and eosinophils in keloid formation was further assessed in a keloid patient single-cell profile (GSE243716) using cutting-edge single-cell analytical frameworks, such as AUCELL, sctenifoldknk, and Bayesprism. In addition, a deep learning algorithm (DrugRefLector) and molecular docking were employed to identify natural compounds targeting the hub gene for the treatment of burn-injury-associated keloid (GSE42270). Finally, *in vitro* assays were conducted to validate expression patterns of the hub gene.

**Results:**

An integrated CE-associated gene signature can indicate keloid formation in burn injury patients. CCL5 can be considered as an up-regulated pathogenic factor and therapeutic target for Icariin, which is involved in keloid pathogenesis.

**Conclusion:**

This is the first study to identify a CA-associated signature in keloid formation among burn injury patients.

## 1 Introduction

Burn injuries represent a severe traumatic insult, initiating a profound multistage pathophysiological response characterized by immediate cell necrosis, a systemic inflammatory surge, and a prolonged reparative phase (1). Keloids are fibroproliferative skin disorders that extend beyond the original wound boundaries, driven by excessive collagen deposition and persistent inflammation (2). The transition from acute burn healing to chronic keloid formation involves complex interactions between immune cells, fibroblasts, and inflammatory signaling pathways, yet predictive biomarkers and targeted therapies remain elusive (3).

Emerging evidence highlights the centrality of the cGAS–STING pathway in sterile inflammation and fibrosis (4). In burn injury, massive cellular damage releases nuclear and mitochondrial DNA, potentially hyperactivating this pathway, thereby perpetuating inflammation and activating fibroblasts (5). Concurrently, eosinophils—traditionally associated with allergic responses and parasite defense—are increasingly recognized for their role in tissue remodeling and fibrosis (6). They infiltrate wound sites and release an arsenal of mediators, including TGF-*β*, IL-4, IL-13, and cationic proteins, which directly stimulate collagen synthesis in fibroblasts (7). In keloids, eosinophil infiltration has been observed, suggesting their contribution to the fibrotic milieu (8). An intriguing, unexplored hypothesis is that the cGAS–STING pathway may regulate eosinophil recruitment or activation postburn, creating a feed-forward loop that drives pathological scarring.

To bridge this knowledge gap, we conducted an integrative multiomics study augmented by artificial intelligence (AI). We hypothesized that a combined molecular signature of cGAS–STING pathway activation and eosinophil activity in the peripheral blood of burn patients could predict subsequent keloid risk and reveal novel therapeutic targets. By sequentially analyzing bulk transcriptomics, constructing machine learning models, examination of findings in single-cell keloid tissue data, and performing *in silico* drug screening, this work established a cGAS–STING pathway and eosinophils (CE)-associated predictive and therapeutic framework for postburn keloid formation. Furthermore, we deciphered the cell-type-specific role and molecular network of CCL5 within keloid eosinophils. Finally, we identified a natural compound with therapeutic potential against this target.

## 2 Material and methods

### 2.1 Source of bulk data

Peripheral blood transcriptomic datasets were retrieved from the Gene Expression Omnibus database using the GEOquery R package and normalized with the Limma package of R. A curated gene list of cGAS–STING pathway was compiled from the GeneCards database (Relevance score >1) (9, 10).

Datasets used in this study include the following:

1. Internal Set (GSE19743, platform GPL570): 63 healthy control and 114 burn patient samples.
2. Training Set (GSE37069, platform GPL570): 37 control and 553 burn injury patient samples.
3. Validation Set (GSE42270, platform GPL570): 16 control and 16 patient samples with keloid.

### 2.2 Identification of differentially expressed genes

In GSE19743, differentially expressed genes (DEGs) between burn patients and controls were identified using the limma package (10). Genes with |log2FC| > 0.5 and *p*_adj_ < 0.05 were considered statistically significant. The resulting DEG list was intersected with the curated cGAS–STING gene list to identify cGAS–STING-associated DEGs. The expression levels of these cGAS-STING-related DEGs were visualized as a heatmap using the pheatmap and ggplot2 packages in R, and GO and KEGG analysis was performed using clusterProfiler in R based on GO and KEGG gene sets retrieved from the MSIGDB database (11, 12).

### 2.3 CIBERSORT and WGCNA analysis

The relative infiltration fraction of 22 immune cell types, including eosinophils, was estimated for each GSE19743 sample using the CIBERSORT algorithm in R (13). A weighted gene coexpression network was then constructed on the normalized expression matrix of GSE28146 using the WGCNA package in R, guided by eosinophil scores derived from CIBERSORT analysis (14). The soft-thresholding power (*β*) was set to achieve scale-free topology fit (14). A topological overlap matrix was generated, and genes were clustered into modules using dynamic tree cutting with a minimum module size of 60 genes (14). The module eigengene showing the highest absolute Pearson correlation (*p*_adj_ < 0.05) with both the burn injury phenotype and the CIBERSORT-derived eosinophil infiltration score was identified as the key eosinophil-related module. Genes from this module were intersected with cGAS–STING-associated DEGs to obtain CE-associated DEGs. A protein–protein interaction (PPI) network was constructed for these genes using the STRING database (confidence score >1.5) and visualized in Cytoscape (v3.10.0) (15). The Molecular Complex Detection (MCODE) plugin was applied to identify the most significant cluster (Degree cutoff = 2, Node score cutoff = 0.2, K-core = 2, Max depth = 100), yielding CE-associated key genes (16).

### 2.4 Machine learning model for diagnostic model construction

In the training and validation sets, we systematically trained and evaluated 132 distinct machine learning models (combinations of 12 base algorithms) using the caret and glmnet packages in R, applying 10-fold cross-validation and calculating the C-Index (17). Model performance was assessed on the training and independent validation sets via receiver operating characteristic (ROC) and precision-recall (PR) curves, with analyses implemented using the pROC package in R (18). A nomogram was constructed using the rms package and calculated with 1,000 bootstrap resamples in the training set (18). The contribution of each key gene to model predictions was quantified using SHAP analysis in R (19). The main contributor was selected as the hub gene for further analysis.

### 2.5 Single-cell analysis

The keloid tissue single-cell RNA-seq dataset (GSE243716) was processed using the Seurat package in R (20). Quality control (QC) filtered out cells with unique feature counts <200 or >6,000, and with mitochondrial gene percentage >20% (20). Data were normalized, scaled, and principal components were calculated. Cells were clustered and visualized using UMAP (20). Cell types were annotated using the SingleR package in R (21). Intercellular communication was analyzed with the CellChat package in R (22). Metabolic pathway activity was inferred using the SCENITH package in R (23). The activity score of the cGAS–STING pathway in each cell was calculated using the AUcell package in R with the cGAS–STING pathway-curated gene set (24). To investigate the network regulated by the hub, a virtual knockout was performed specifically on the eosinophil cluster using the scTenifoldKnk tool in R, with genes having an adjusted *p*-value (FDR) < 0.05 considered significantly perturbed (25). KEGG, GO and DO enrichment analyses of these dysregulated genes were conducted using the R packages clusterProfiler and DOSE, with reference to gene sets sourced from the MSIGDB database (12, 26). To deconvolute eosinophil-specific signals in bulk data, the eosinophil cluster from GSE243716 was used as a reference with the BayesPrism framework in R to estimate eosinophil-specific features in GSE42270 (27). The correlation between the inferred eosinophil activity scores and keloid progression was assessed using ROC analysis via the pROC package in R (28). Differentiation patterns were analyzed using the monocle2 package in R (29).

### 2.6 Deep learning for drug screening

Potential therapeutic natural compounds were screened using an integrated strategy. First, the hub gene was used as a target to query the Traditional Chinese Medicine Systems Pharmacology (TCMSP) database (30). Second, the DrugReflector deep learning framework was applied to the gene expression profile of GSE42270 to predict compounds that could reverse keloid to healthy status (31). The optimal compound was prioritized. Molecular docking of the top candidate (Icariin, Compound CID: 5318997) with the human CCL5 protein (PDB ID:707F) was performed using the CB-DOCK2 platform. Posing with a Vina score (-kcal/mol) < −7.0 were considered to indicate strong binding potential (32).

### 2.7 Cell lines and culture conditions

The human eosinophilic cell line EOL-1 was purchased from SUNCELL (China) and cultured in RPMI-1640 medium (Gibco, Germany) supplemented with 10% FBS (Gibco, Germany) and 1% penicillin–streptomycin (Gibco, Germany). To model a keloid-associated inflammatory microenvironment *in vitro*, cells were treated with a combination of 10 ng/mL IL-4 and 10 ng/mL IL-13 (Vazyme, China). Untreated cells served as controls.

### 2.8 RNA extraction and q-RT-PCR

Total RNA was extracted using TRIzol (Takara, China). cDNA was synthesized using the HiScript III RT SuperMix (Vazyme, China). qRT-PCR was performed with Universal SYBR qPCR Master Mix (Vazyme, China). CCL5 expression was normalized to GAPDH using the 2^(-*ΔΔ*Ct) method. The following primer sequences were used in this study:

CCL5:

F: 5′-CAGTCGTCTTTGTCACCCGAA-3′,

R:5′-GCAAGCAGAAAGAGCAGAAGCA-3′

GAPDH:

F:5′-GTCTCCTCTGACTTCAACAGCG-3′,

R: 5′-ACCACCCTGTTGCTGTAGCCAA-3′

### 2.9 Statistical analysis

For bioinformatics analyses, an adjusted *p*-value (FDR) < 0.05 or a two-sided *p*-value <0.05 was considered significant. For *in vitro* data, results from three independent experiments are presented as mean ± SD. Comparisons between two groups were analyzed using an unpaired Student's *t*-test. Values of *P* < 0.05 were considered statistically significant.

## 3 Results

### 3.1 Identification of cGAS–STING signal-related DEGs in burn injury patients

**Figure 1:**
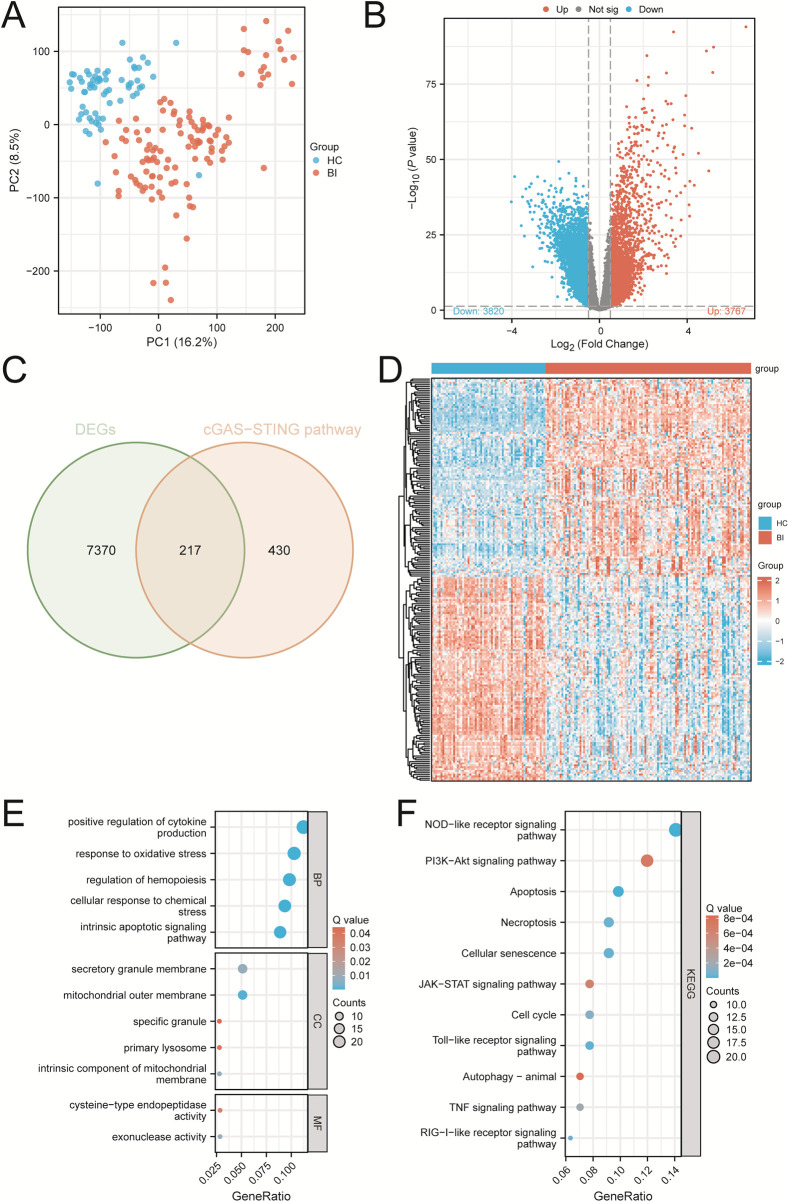
Identification of cGAS–STING pathway-associated molecular patterns in burn injury patients. **(A)** PCA plot of the internal dataset (GSE19743) shows transcriptomic separation between burn injury patients and healthy controls. **(B)** Volcano plot displaying differentially expressed genes (DEGs). **(C)** Venn diagram illustrating the intersection between the DEG list and a curated cGAS–STING pathway gene lists. **(D)** Heatmap displaying the expression patterns of the cGAS–STING-associated DEGs across burn and control samples. **(E,F)** KEGG and GO bar plots of the top enriched pathways for cGAS–STING-associated DEGs.

In GSE19743, distinct transcriptomic profiles were observed between patients and controls (Figure 1A). Differential expression analysis identified 7,587 significant DEGs (|log2FC|>0.5, padj < 0.05) (Figure 1B). Intersection with the cGAS–STING gene list yielded 217 cGAS–STING-associated DEGs (Figure 1C). A heatmap confirmed their expressions pattern in burn injury patients compared with controls (Figure 1D). Enrichment analysis of these genes highlighted significant involvement in inflammation-related pathways and cellular response to stress (Figure 1E).

### 3.2 Identification of eosinophil-associated molecular signature in burn injury patients

**Figure 2:**
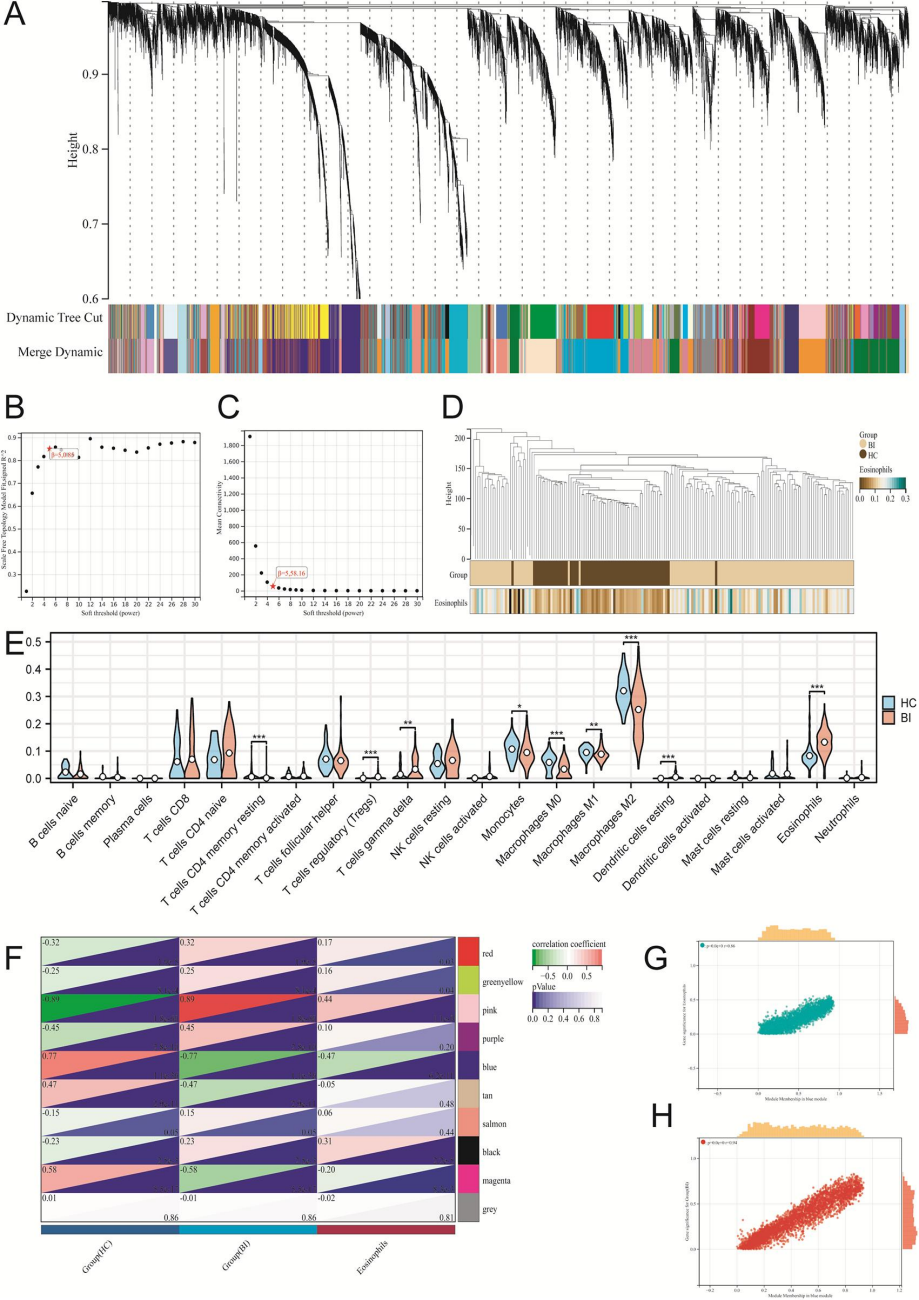
Identification of eosinophil-associated coexpression modules in burn injury patients. **(A)** Cluster dendrogram of genes from GSE19743. **(B,C)** Analysis of network topology for various soft-thresholding powers (*β*) in WGCNA. **(D)** Cluster dendrogram of genes from GSE28146, with colors indicating distinct coexpression modules identified by dynamic tree cutting. **(E)** Box plot comparing the CIBERSORT-estimated eosinophil infiltration fraction between burn patients and controls in the GSE28146. **(F–H)** Module–trait relationship heatmap. The module eigengene of the blue module demonstrated the highest significant positive correlation with both the burn injury phenotypes.

In GSE19743, CIBERSORT analysis revealed a significantly elevated eosinophil infiltration fraction in burn patient samples compared with controls (Figure 2E). WGCNA constructed a scale-free coexpression network and identified 10 distinct modules (Figures 2A–D,F). The “blue” module (containing 3,508 genes) exhibited the strongest positive correlation with both the burn injury status and eosinophil scores (Figures 2G,H).

### 3.3 Identification of diagnostic model for forecasting keloid formation in burn injury patients

**Figure 3:**
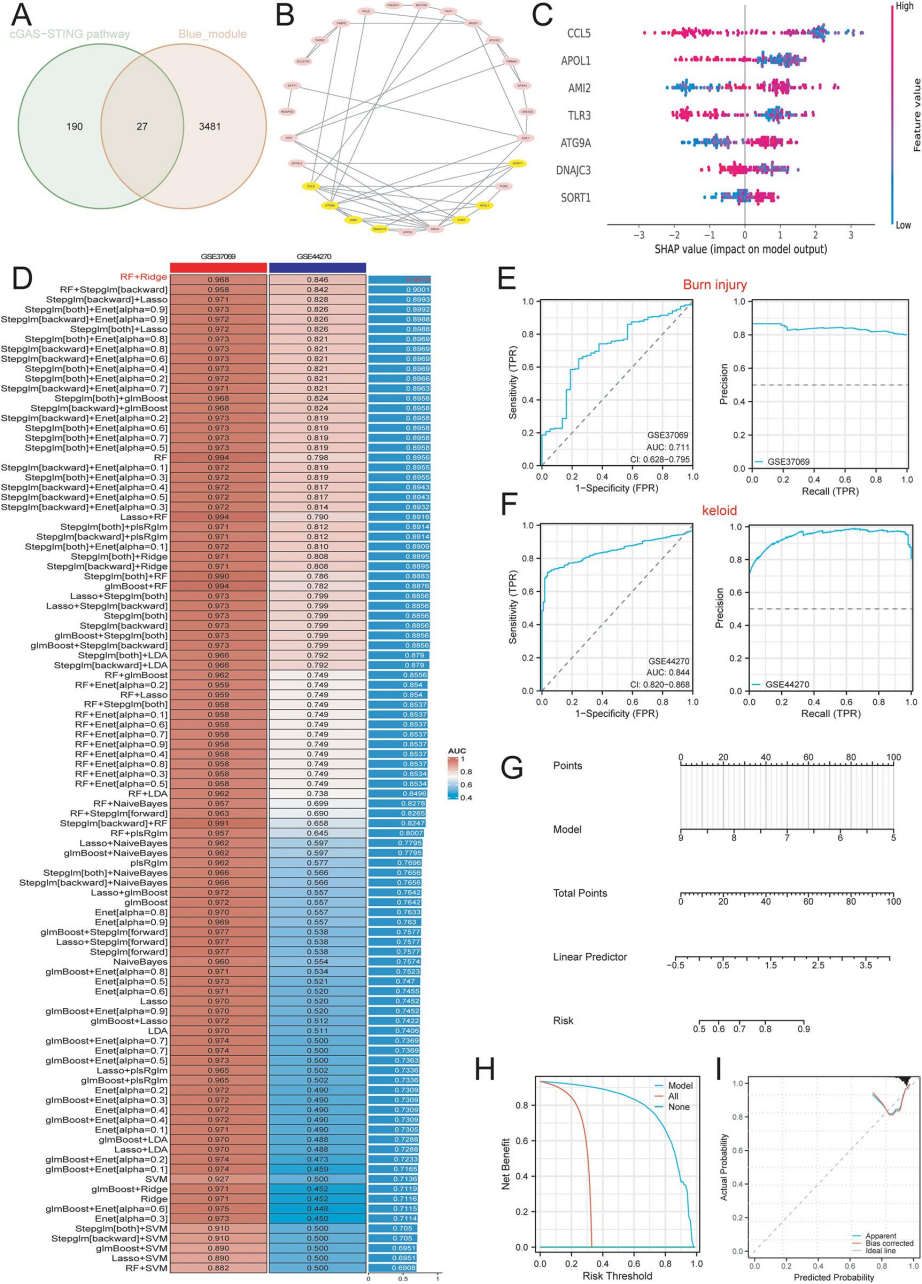
Construction and examination of a CE-associated ensemble diagnostic model for predicting keloid risk in burn injury patients. **(A)** Identification of 27 CE-associated DEGs. **(B)** PPI network of the 27 CE-associated DEGs constructed using STRING. **(C)** SHAP summary plot showing the contribution of the seven key genes to the model output, with CCL5 identified as the top contributor. **(D)** Machine learning models from the evaluation of 132 algorithm combinations in the training and validation sets. **(E)** ROC and PR curves in training set. **(F)** ROC and PR curves in validation set. **(G–I)** Nomogram and calibration analysis of model.

Intersection of the 217 cGAS–STING-associated DEGs and the 3,508 blue-module genes yielded 27 CE-associated DEGs (Figure 3A). A PPI network of these 27 genes and subsequent MCODE analysis identified a key cluster of 7 genes (Figure 3B). In the training and validation sets, systematic evaluation of 132 machine learning models identified Random Forest with Ridge regression penalty (RSF + Ridge) as the optimal model, achieving the highest cross-validated C-index (Figure 3D). This model demonstrated excellent diagnostic performance in both the training set and independent validation set, indicating that this model can potentially predict keloid formation in burn injury patients with favorable accuracy and efficacy (Figures 3E–I). SHAP analysis identified CCL5 as the gene with the highest mean absolute SHAP value, underscoring its paramount contribution to the model's predictive power (Figure 3C).

### 3.4 Single-cell atlas of eosinophils in keloid after burn injury

**Figure 4:**
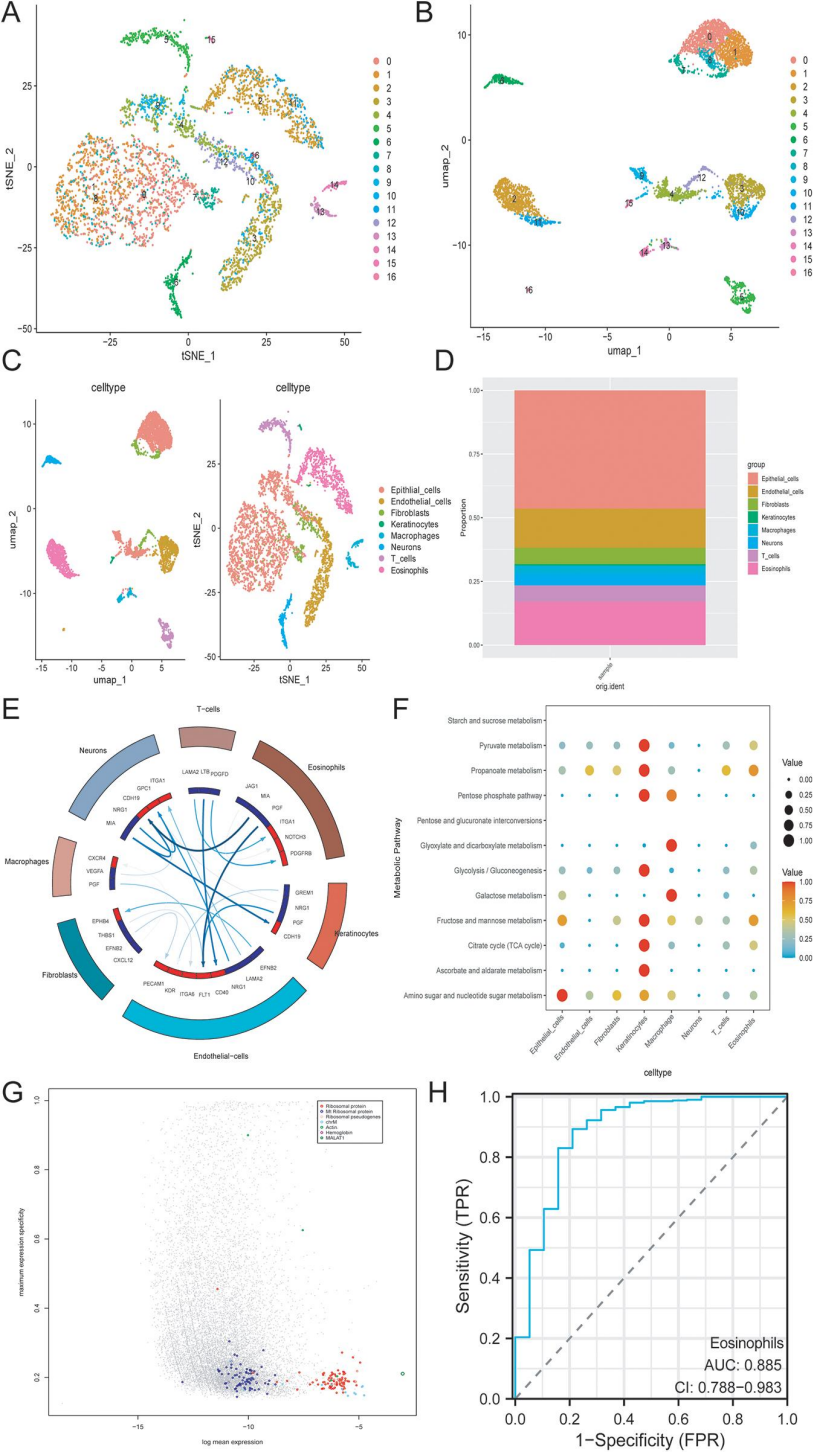
Single-cell landscape and eosinophil implications in keloid tissue. **(A,B)** UMAP and t-SNE visualization of clusters. **(C,D)** UMAP and t-SNE visualization of cell types. **(E)** Circle plot of inferred intercellular communication networks using CellChat, highlighting enhanced incoming signaling to fibroblasts, primarily mediated by MIF and ANNEXIN pathways. **(F)** Heatmap comparing metabolic pathway activity scores. **(G,H)** Bayesprism analysis.

Analysis of keloid tissue single-cell data (GSE243716) after QC identified 8,542 high-quality cells annotated into 16 cell clusters and 8 major cell types, including a distinct population of eosinophils (Supplementary Figures S1A–D, and Figures 4A,B). CellChat analysis revealed strong incoming communication signals from eosinophil to fibroblasts (Figure 4E). Metabolic profiling showed elevated propanoate metabolism activity in keloid eosinophils (Figure 4F). BayesPrism indicated that eosinophil features were associated with keloid formation and progression (Figures 4G,H).

### 3.5 CCL5 molecular mechanism estimation in keloid eosinophils

**Figure 5:**
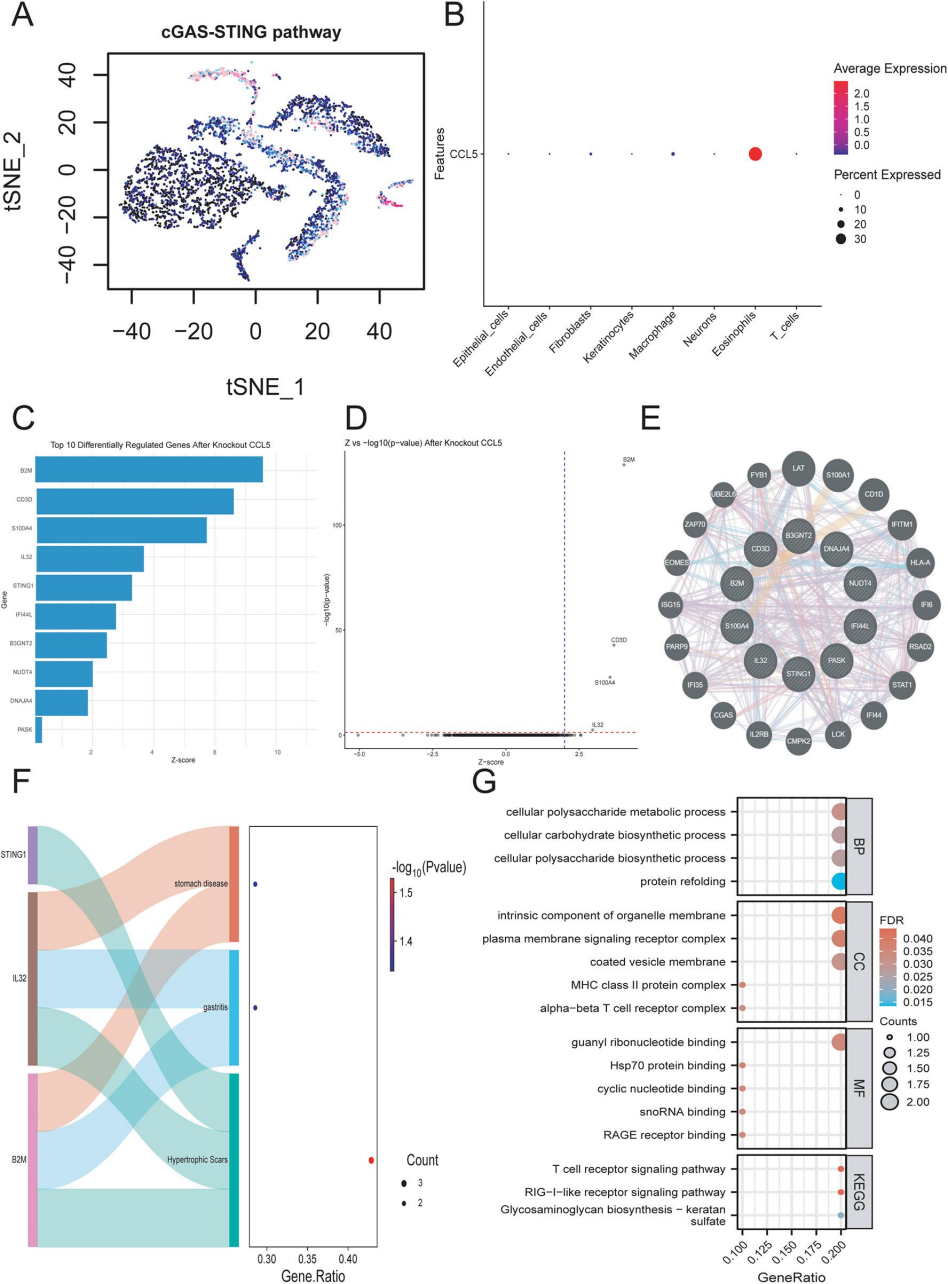
Molecular mechanisms of CCL5 in keloid-infiltrating eosinophils. **(A)** AUCELL analysis of cGAS–STING pathway. **(B)** Distribution of CCL5 in eosinophils. **(C,D)** Network perturbation graph generated by the scTenifoldKnk tool following a virtual knockout of CCL5 within the eosinophil cluster. **(E–G)** Molecular characters of perturbed genes. **(H,I)** Monocle2 analysis.

AUCell scoring demonstrated that cGAS–STING pathway activity was significantly enriched within the eosinophil cluster (Figure 5A). CCL5 expression was confirmed to be highly specific to the eosinophil cluster in keloid tissue (Figure 5B). Typically, KO of CCL5 can affect the expression of STING (Figures 5C–E). Virtual knockout of CCL5 within the eosinophil cluster using scTenifoldKnk identified perturbed genes involved in scar formations and inflammation-related pathways (Figures 5F,G). Next, monocle2 quantified expression patterns in eosinophil differentiation (Figures 5H,I). These findings indicate that CCL5 was associated with keloid formation in eosinophils by affecting the cGAS–STING pathway.

### 3.6 Therapeutic agent screening for treatment of keloid

**Figure 6:**
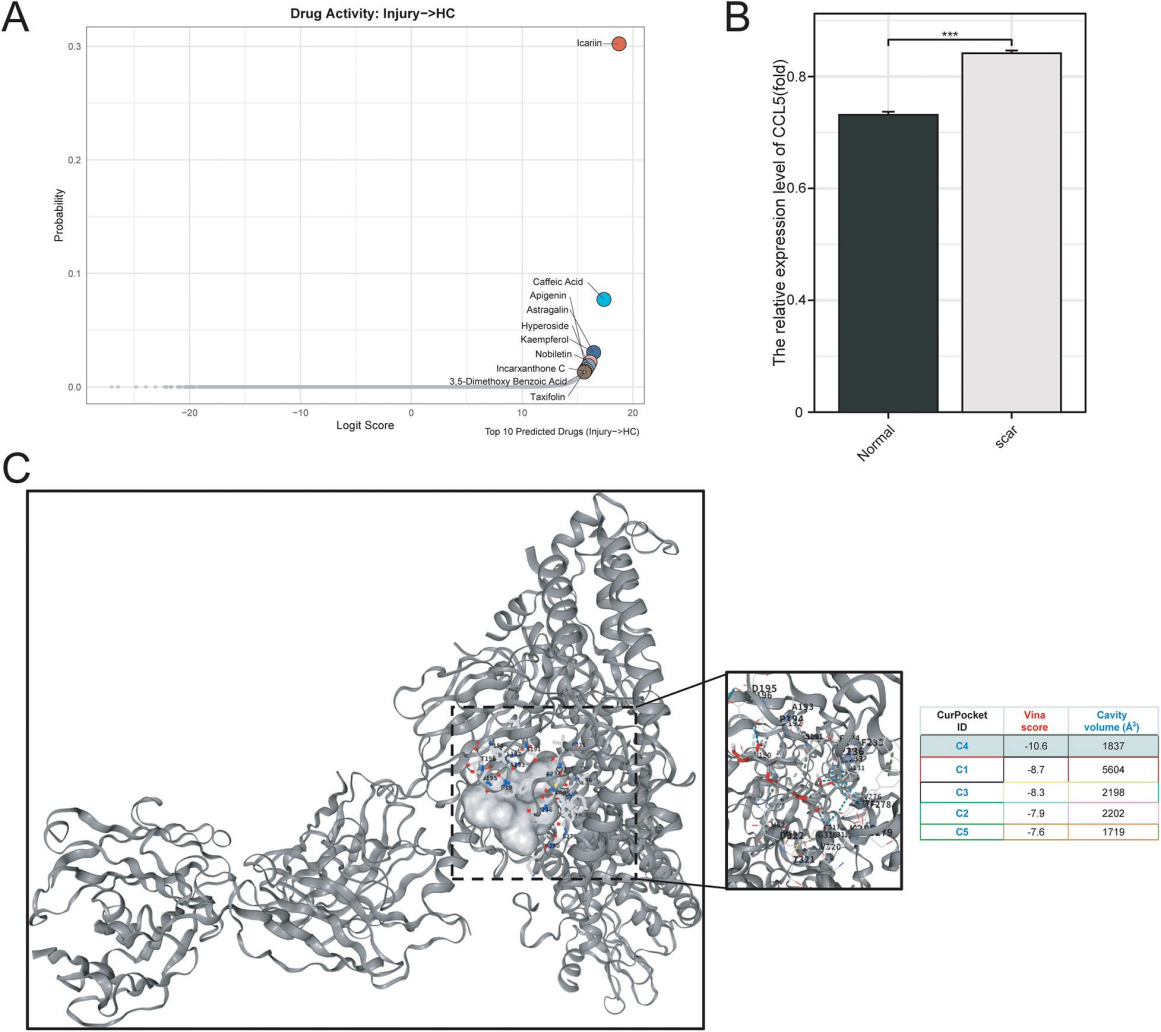
Deep learning-driven therapeutic screening and *in vitro* validation. **(A)** Schematic workflow integrating network pharmacology (TCMSP database) and the DrugReflector deep learning framework to prioritize therapeutic natural compounds targeting the CE-associated signature and CCL5. **(B)** qRT-PCR validation of CCL5 mRNA expression in EOL-1 modeled under a keloid-mimicking inflammatory microenvironment. **(C)** Molecular docking validation.

Integrated screening via the TCMSP database and the DrugReflector model on the GSE42270 signature prioritized Icariin as the top natural compound targeting the CE-signature and CCL5 (Figure 6A). Molecular docking predicted stable binding of Icariin to the CCL5 protein with a favorable Vina score of −10.6 kcal/mol (Figure 6C). *In vitro* validation using the keloid-mimicking eosinophil model (EOL-1 cells treated with IL-4/IL-13/CCL5) confirmed significant upregulation of CCL5 mRNA compared to untreated control cells (Figure 6B).

## 4 Discussion and conclusion

This study establishes a novel predictive association between cGAS–STING pathway activation, eosinophil function, and keloid risk following burn injury. By integrating bulk transcriptomics of burn injury patients with single-cell analysis of keloid tissue, we defined a robust CE-associated molecular signature, validated a high-accuracy diagnostic model, and nominated CCL5 as the central hub gene within eosinophils.

Our findings suggested that CCL5 in eosinophil is associated with cGAS–STING pathway regulation and keloid formation. CCL5 is a potent chemokine for various leukocytes, including eosinophils (33). The cGAS–STING pathway is a potent inducer of type I interferon (IFN) and downstream interferon-stimulated genes (34). Notably, IFN-*γ* and type I IFNs are known transcriptional activators of CCL5 (35). CCL5 exerts its biological effects primarily by binding to its receptors, notably CCR1, CCR3, and CCR5. CCR3 is highly expressed on eosinophils, making them prime targets for CCL5-mediated chemotaxis (36). Prior studies in allergic inflammation models have consistently demonstrated that CCL5–CCR3 signaling is a key driver of eosinophil recruitment to sites of inflammation (37). Beyond chemotaxis, CCL5 also contributes to eosinophil activation and degranulation, potentiating the release of pro-fibrotic mediators such as TGF-*β* and cationic proteins (38). However, the mechanisms of CCL5 in regulating eosinophil have not yet been reported.

In conclusion, we decoded a novel pathogenic axis in postburn keloid formation, centered on cGAS–STING pathway-associated CCL5 expression within eosinophils. This work provides novel multigene signature for predicting keloid risk in burn injury patients and highlights natural therapeutic strategies. However, there are still limitations in our study. For example, our study relied on different datasets from burn injury patients and keloid patients, which could cause batch effects on the predictive and therapeutic model. Future studies should focus on validating model accuracy in clinical studies of burn injury patients with keloid formation to enhance the robustness. Besides, the regulatory function of CCL5 within the eosinophil cGAS–STING signaling axis in keloid development needs further verification using preclinical keloid models and clinical burn injury samples, so as to strengthen the reliability of our results. In addition, the immune mechanisms of eosinophils in keloid formation of burn injury patients, particularly the implications of interacting with macrophages (well-known regulators of wound healing and fibrosis), should be further clarified in preclinical and clinical trials. Furthermore, the precise upstream mechanisms linking burn injury to cGAS–STING activation in eosinophils, and the detailed downstream effects of CCL5 blockade on fibroblast activity *in vivo*, require further experimental investigation.

## Data Availability

The transcriptomic datasets used in the present study are publicly available in the Gene Expression Omnibus (GEO) database (https://www.ncbi.nlm.nih.gov/geo/) under the following accession numbers: GSE28146, GSE37069, GSE42270, and GSE243716. The cGAS–STING pathway-related gene list was retrieved from the GeneCards database (https://www.genecards.org/) with a relevance score threshold of >1. The Traditional Chinese Medicine Systems Pharmacology (TCMSP) database (https://old.tcmsp-e.com/tcmsp.php) was used for natural compound screening targeting the hub gene. All original experimental data generated from the *in vitro* cell assays (qRT-PCR) in this study are available from the corresponding author upon reasonable request. The R code used for bioinformatics and machine learning analyses in this study is also available from the corresponding author upon reasonable request.
