# Supplementary material for: Decoding the cGAS–STING–eosinophils predictive and natural therapeutic molecular signature in burn injury progression and keloid formation: insights from artificial intelligence-driven multiomics
Source: Front Surg. 2026 May 29;13:1846856. doi: 10.3389/fsurg.2026.1846856 (PMC13261910; doi:10.3389/fsurg.2026.1846856)
Supplement: Supplementary file 1 [file Supplementaryfile1.docx]

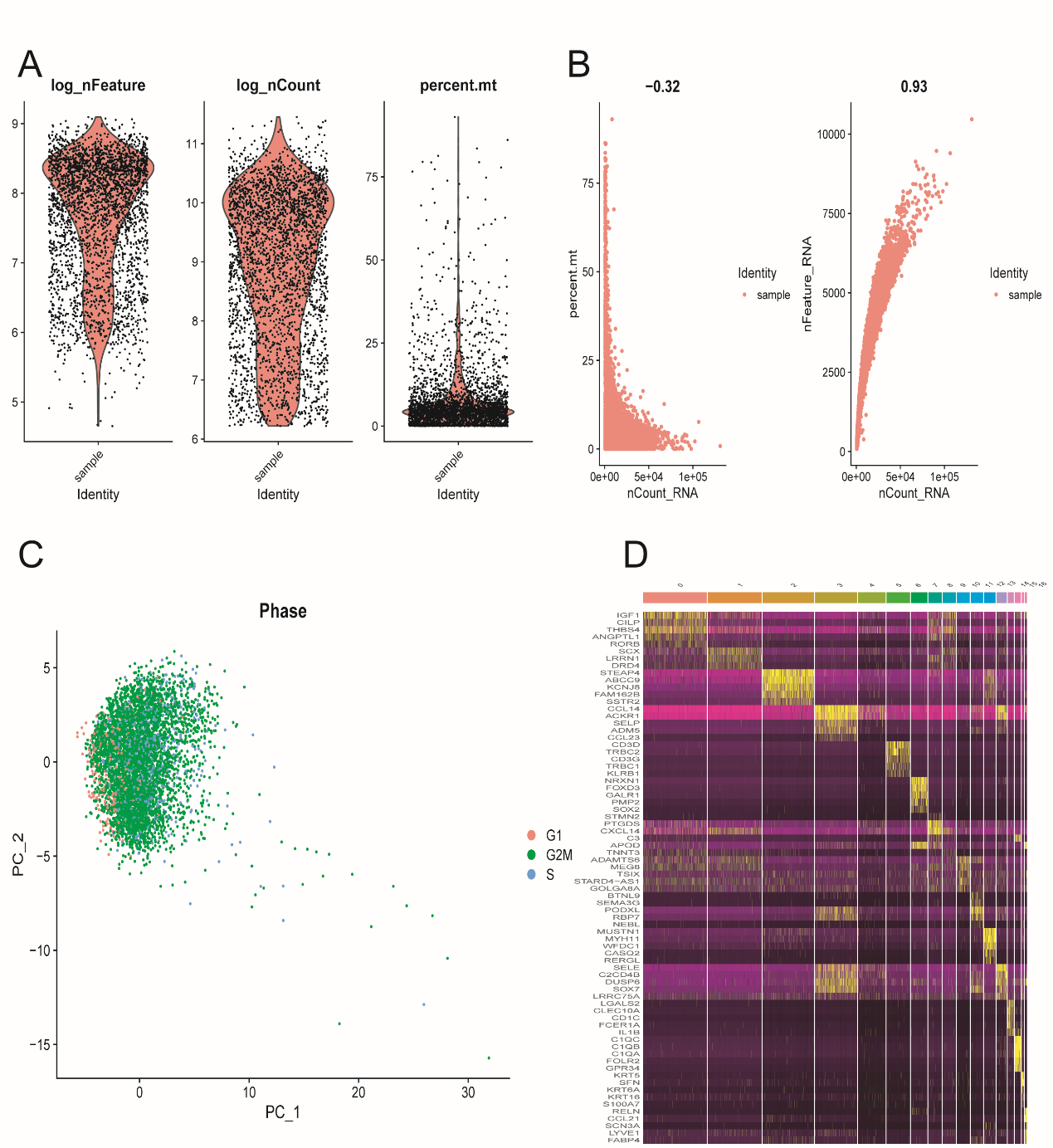


**Figure S1: Pre-processing of single-cell data. (A-C)** QC metrics of single-cell data. **(D)** Markers of various clusters.
