# Supplementary figures and images for: Decoding the cGAS–STING–eosinophils predictive and natural therapeutic molecular signature in burn injury progression and keloid formation: insights from artificial intelligence-driven multiomics
Source: Front Surg. 2026 May 29;13:1846856. doi: 10.3389/fsurg.2026.1846856 (PMC13261910; doi:10.3389/fsurg.2026.1846856)

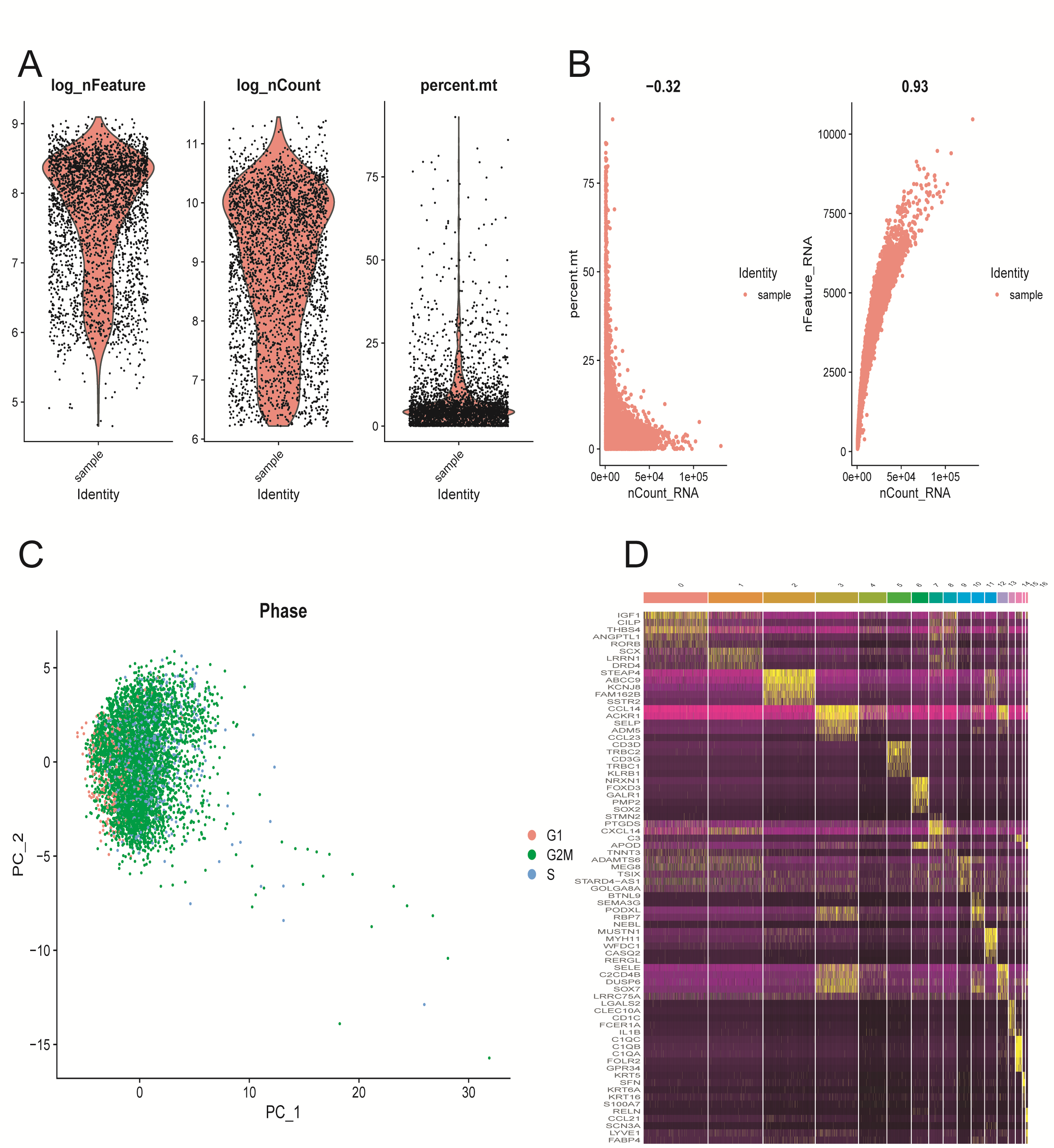

Supplement: Supplementary file 2 [file Image1.tif]
